# In Vitro and In Vivo Degradation of Photo‐Crosslinked Poly(Trimethylene Carbonate‐*co*‐ε‐Caprolactone) Networks

**DOI:** 10.1002/mabi.202300364

**Published:** 2023-11-15

**Authors:** Bas van Bochove, Jan J. Rongen, Gerjon Hannink, Jukka V. Seppälä, André A. Poot, Dirk W. Grijpma

**Affiliations:** ^1^ Advanced Organ Bioengineering and Therapeutics Faculty of Science and Technology University of Twente Drienerlolaan 5 Enschede 7522 NB The Netherlands; ^2^ Polymer Technology, School of Chemical Engineering Aalto University Otakaari 1 B Espoo 02150 Finland; ^3^ Orthopedic Research Laboratory Radboud University Medical Center Geert Grooteplein Zuid 10 Nijmegen 6525 GA The Netherlands; ^4^ Department of Medical Imaging Radboud University Medical Center Geert Grooteplein Zuid 10 Nijmegen 6525 GA The Netherlands

**Keywords:** degradation, networks, photo‐crosslinking, poly(trimethylene carbonate), surface erosion

## Abstract

Three‐armed poly(trimethylene carbonate) (PTMC) and poly(trimethylene carbonate‐*co*‐Ɛ‐caprolactone) (P(TMC‐*co*‐ε‐CL)) macromers with molecular weights of approximately 30 kg mol^−1^ are synthesized by ring‐opening polymerization and subsequent functionalization with methacrylic anhydride. Networks are then prepared by photo‐crosslinking. To investigate the in vitro and in vivo degradation properties of these photo‐crosslinked networks and assess the effect of ε‐caprolactone content on the degradation properties, PTMC networks, and copolymer networks with two different TMC:ε‐CL ratios are prepared. PTMC networks degraded slowly, via an enzymatic surface erosion process, both in vitro and in vivo. Networks prepared from P(TMC‐*co*‐ε‐CL) macromers with a 74:26 ratio are found to degrade slowly as well, via a surface erosion process, albeit at a higher rate compared to PTMC networks. Increasing the ε‐CL content to a ratio of 52:48, resulted in a faster degradation. These networks lost their mechanical properties much sooner than the other networks. Thus, PTMC and P(TMC‐*co*‐ε‐CL) networks are interesting networks for tissue engineering purposes and the exact degradation properties can be tuned by varying the TMC:ε‐CL ratio, providing researchers with a tool to obtain copolymer networks with the desired degradation rate depending on the intended application.

## Graphical abstract

The degradation of photo‐crosslinked networks prepared from poly(trimethylene carbonate‐*co*‐ε‐caprolactone) is evaluated. In vitro, network degradation is characterized by enzymatic surface erosion, in particular that of the 75:25 copolymer network. In vivo, a similar trend is seen, albeit at a lower rate as compared to in vitro.

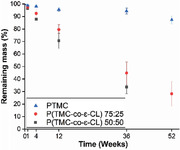

## 1 Introduction

Biodegradable tissue engineering scaffolds are used to induce the (re)generation of tissues in vitro and in vivo, often in combination with cells and/or biologically active components.^[^ ^1^ ^]^ Scaffolds are porous implants intended to temporarily support cells and formed tissues. Ideally, such scaffolds have high porosity, good pore interconnectivity, and optimal pore sizes for their intended application.^[^ ^2^, ^3^, ^4^ ^]^ Tissue engineering scaffolds need to be biocompatible, biodegradable, and have mechanical properties that are compatible with those of the tissues that they replace.^[^ ^2^, ^5^, ^6^ ^]^

Biodegradable polymers with a low glass transition temperature (T_g_) form an interesting class of biomaterials for use in biomedical applications^[^ ^7^, ^8^, ^9^, ^10^, ^11^, ^12^ ^]^ as the low T_g_ results in materials with low stiffness. For regenerative medicine and soft tissue engineering, it would be advantageous to use biocompatible crosslinked elastomeric scaffolds as they withstand dynamic loading, are form‐stable and their surfaces allow for cell attachment and proliferation.^[^ ^13^, ^14^ ^]^

By functionalizing biodegradable oligomers with low T_g_ with (meth)acrylate end‐groups, (photo)‐crosslinkable macromers with relatively low viscosity can be obtained.^[^ ^15^, ^16^ ^]^ Subsequent photo‐crosslinking results in form‐stable, creep‐resistant elastomeric networks.^[^ ^14^ ^]^ Additionally, this allows for the preparation of designed tissue engineering scaffolds by 3D printing techniques such as stereolithography.^[^ ^17^, ^18^ ^]^ 3D printing techniques hold several advantages over more conventional techniques such as particulate leaching and phase‐separation and allow for the preparation of scaffolds with optimal properties regarding: i) pore structure and connectivity, ii) geometry, iii) mechanical properties, iv) cell‐seeding efficiency, and v) transport of nutrients and metabolites.^[^ ^17^, ^19^ ^]^ Furthermore, 3D printing techniques allow for the preparation of patient‐specific implants.^[^ ^20^, ^21^, ^22^ ^]^

Poly(trimethylene carbonate) (PTMC) is an amorphous, flexible, biodegradable and biocompatible polymer.^[^ ^14^, ^23^ ^]^ High molecular weight PTMC was found to degrade via surface erosion.^[^ ^24^ ^]^ By photo‐crosslinking methacrylate‐functionalized PTMC oligomers, tough, and tear‐resistant networks can be obtained. Networks prepared from PTMC macromers with molecular weights in excess of approximately 10 kg mol^−1^ had high tensile strengths, toughness, suture retention strengths, and tear resistance.^[^ ^14^ ^]^ The strength and toughness of these networks increased with the increasing molecular weight of the macromers used to prepare the networks. To prepare flexible, elastic, and load‐bearing tissue engineering scaffolds such as meniscus implants and intervertebral discs, networks prepared from PTMC macromers with relatively high molecular weights are therefore preferred.^[^ ^25^ ^]^ Meniscus tissue, for example, has compressive aggregate moduli ranging from 11.5 to 150 kPa depending on the amount of strain applied.^[^ ^26^ ^]^

Previously, we reported on the in vitro and in vivo degradation of PTMC networks prepared from PTMC macromers with different molecular weights.^[^ ^27^ ^]^ These networks degraded via enzymatic surface erosion, but the degradation rate was very slow, with only 1.7%–3.2% mass loss after 26 weeks in vitro and only 0.5%–3.6% mass loss in vivo after 36 weeks. The networks prepared from the higher molecular weight macromers (18 and 27 kg mol^−1^) had somewhat higher rates of mass loss compared to the networks prepared from the lower molecular weight macromers (13 kg mol^−1^). Other in vitro^[^ ^28^ ^]^ and in vivo^[^ ^29^ ^]^ studies have further confirmed the low degradation rate of photo‐crosslinked PTMC networks.

To tune the degradation rate, networks can be prepared from copolymeric macromers of TMC with other monomers such as D,L‐lactide or ε‐caprolactone ε‐CL.^[^ ^30^, ^31^, ^32^ ^]^ Poly(ε‐caprolactone) (PCL) is a highly biocompatible semicrystalline polymer with a T_g_ of around −60 ⁰C and a melting point close to 65 ⁰C.^[^ ^33^, ^34^, ^35^ ^]^ Photo‐crosslinking of PCL macromers leads to amorphous, rubber‐like networks.^[^ ^36^ ^]^ These networks show a decrease in tensile modulus and an increase in elongation at break with increasing molecular weight of the macromer. Networks prepared from low molecular weight P(TMC‐*co*‐ε‐CL) macromers (7800 g mol^−1^) were found to degrade via surface erosion.^[^ ^31^ ^]^

In this study, we prepared (co)polymeric network films by photo‐crosslinking methacrylated PTMC and P(TMC‐*co*‐ε‐CL) oligomers of relatively high molecular weight containing different amounts of ε‐CL. These networks were subjected to enzymatic degradation by cholesterol esterase in vitro, and subcutaneous degradation in vivo in rats. Cholesterol esterase was chosen for the in vitro degradation as earlier studies showed that cholesterol esterase degrades PTMC networks in vitro.^[^ ^27^, ^37^, ^38^ ^]^ The degradation behavior was investigated by monitoring the changes in mass and thickness of the networks over time, and the tissue response to the implantation of the networks.

## 2 Experimental Section

### 2.1 Materials

Trimethylene carbonate (TMC) was provided by Huizhou Foryou Medical Devices Co. (China). ε‐CL was purchased from Acros Organics (Germany), dried over calcium hydride (Sigma, USA), and distilled before use. Trimethylol propane (TMP), tin(II) 2‐ethylhexanoate (Sn(Oct)_2_), deuterated chloroform, methacrylic anhydride, triethylamine, and hydroquinone were obtained from Sigma. Dichloromethane (DCM) was purchased from VWR Chemicals (France), dried over calcium hydride, and distilled before use. Ethanol and propylene carbonate were obtained from Merck (Germany). Omnirad TPO‐L was provided by IGM Resins (the Netherlands). Phosphate‐buffered saline (PBS), sodium azide (NaN_3_), and cholesterol esterase were purchased from Sigma. Orasol Orange was obtained from Ciba Specialty Chemicals.

### 2.2 Synthesis and Characterization of PTMC and P(TMC‐co‐ε‐CL) Macromers

Three‐armed PTMC and random P(TMC‐*co*‐ε‐CL) oligomers were synthesized by ring‐opening polymerization of TMC and ε‐CL using TMP as initiator and Sn(Oct)_2_ (5×10^−5^ mol mol^−1^ monomer) as catalyst. The monomers and initiator were charged in a silanized three‐necked flask and polymerized for 3 days at 130 ⁰C under an argon atmosphere. For the copolymers, monomer molar ratios of 75:25 and 50:50 (TMC:CL) were used. Furthermore, by controlling the initiator to monomer ratio, oligomers with a targeted molar mass (M_n_) of 30 kg mol^−1^ were prepared (using TMP:TMC:CL molar ratios of 1:294.1:0 for PTMC, 1:213.9:71.3 for P(TMC‐*co*‐ε‐CL) 75:25, and 1:138.6:138.6 for P(TMC‐*co*‐ε‐CL) 50:50). The *M*_n_ of the obtained oligomers and their composition were determined by ^1^H‐NMR (Varian Inova 400 MHz, Brüker, Germany) using deuterated chloroform solutions. The *M*_n_ of the PTMC oligomer was determined by comparing the integral values of the TMC ‐CH_2_‐ peaks at δ 2.05 and 4.24 ppm to the value of the ‐CH_3_ peak of the TMP initiator at δ 0.92 ppm. The molar ratio and the *M*_n_ of the P(TMC‐*co*‐ε‐CL) oligomers were determined by comparing the integral values of the ε‐CL‐CH_2_‐ peaks at δ 1.38, 1.65, and 2.31 ppm and the TMC ‐CH_2_‐ peak at δ 2.05 ppm to the value of the –CH_3_ peak of the TMP initiator at δ 0.92 ppm.

The oligomers were dissolved in dried DCM (2 ml/g monomer) and subsequently functionalized under argon atmosphere by reaction with methacrylic anhydride (7.5 mol mol^−1^ oligomer) in the presence of triethylamine (7.5 mol mol^−1^ oligomer) and 0.1 wt.% hydroquinone. After 5 days at room temperature, the methacrylate‐functionalized oligomers (macromers) were precipitated in cold ethanol and dried under vacuum for 1 week. The degree of functionalization (DF) of all macromers was determined by ^1^H‐NMR by comparing the integral values of the ‐C=CH_2_ peaks that appeared at δ 5.57 and 6.11 ppm to the value of the –CH_3_ peak of the TMP initiator at δ 0.92 ppm.

### 2.3 Preparation and Characterization of Photo‐crosslinked Networks

The photo‐crosslinked networks were prepared in a UV‐crosslinking cabinet (365 nm, 8–10 mW cm^2^, Ultralum, USA). The macromers were dissolved in propylene carbonate at 60 wt.%. Subsequently, 5 wt.% TPO‐L photo‐initiator and 0.15 wt.% Orasol orange (both relative to the macromer) were added. To obtain networks with a thickness of approximately 500 µm, films were casted at 850 µm thickness, taking into account the shrinkage during extraction. The resins had a temperature of 70 ⁰C and the casted films were irradiated for 30 min under a nitrogen atmosphere. The obtained crosslinked network films were extracted by immersion in propylene carbonate/ethanol mixtures, starting at 50:50 vol.%. The extraction medium was changed daily, increasing the ethanol content by 10 vol.% daily. The network films were subsequently dried until constant weight.

Differential Scanning Calorimetry (DSC) was used to determine the T_g_ of extracted and dried networks. DSC measurements were done using a Pyris 1 DSC (Perkin Elmer, USA) with samples weighing 5–10 mg. The samples were heated to 100 ⁰C at a rate of 10 ⁰C min^−1^ and then cooled to −100 ⁰C min^−1^ at a rate of 200 ⁰C min^−1^. After 5 min at −100 ⁰C the samples were heated to 100 ⁰C at a rate of 10 ⁰C min^−1^. The T_g_s of the samples were determined from the second heating scan.

### 2.4 In Vitro Degradation

In vitro degradation of P(TMC‐*co*‐ε‐CL) photo‐crosslinked networks was investigated using disc‐shaped samples with a diameter of 1 cm and an approximate thickness of 500 µm. A network of the PTMC homopolymer prepared from a similar molecular weight was included as a reference for the degradation. Samples were placed in well plates containing 1 mL of either PBS (pH7.4) containing 0.02 wt.% NaN_3_ as a bactericide or a solution of 20 µg ml^−1^ cholesterol esterase in PBS containing 0.02 wt.% NaN_3_. The samples were incubated at 37 ⁰C and the degradation solutions were changed twice per week. At predetermined time points the samples were rinsed, dried to constant weight at 37 ⁰C and evaluated with regard to their mass and thickness. Degradation of each network in the different degradation media was performed in triplicate per time point. Additionally, the mechanical stability of the networks was assessed qualitatively.

### 2.5 In Vivo Degradation and Tissue Response

The in vivo degradation of and tissue response to the homopolymer and copolymer networks were assessed subcutaneously in rats. All procedures performed on animals were done according to international guidelines on animal experiments and approved by the local committee for the care and use of laboratory animals (DEC‐No.: 2015‐0087). Twenty rats (10 male, 10 female, Sprague Dawley, Charles River, Germany) were used, weighing between 335 and 464 g at the start of the experiment. The animals were housed 2–4 rats per cage, received water and pelleted diet ad libitum, and were placed within the same humidity‐ and temperature‐controlled animal room facility with 12 h light/dark cycles.

A sample size calculation resulted in four implants per network per time point for the evaluation of degradation. Three types of implants were evaluated, composed of the three networks: PTMC, P(TMC‐*co*‐ε‐CL) 74:26, and P(TMC‐*co*‐ε‐CL) 52:48. The implants were rectangular films of 20×10 mm with an approximate thickness of 500 µm. Rats underwent operation under general anesthesia and were euthanized using carbon dioxide after 1, 4, 12, 26, and 52 weeks, respectively. Implants were inserted into four noncommunicating, subcutaneous pockets (one implant per pocket) that were made on the back of each rat. The implants from three of those pockets were used for the assessment of implant degradation. Implants in the fourth pocket were used for the evaluation of tissue response (three implants per time point, one of each network). A computer‐based pseudo‐random number generator was used to randomize the distribution of the different implants over the different pockets and over the different animals per group. Before implantation, 70% ethanol was used to decontaminate the implants.

*Remaining mass and thickness*: Before implantation, the implants were dried until constant weight at 40 °C. Subsequently, their initial dry mass and thickness were determined. The thickness of the implants was measured at five predefined locations per implant (at the four edges and its center) and the average value was used as implant thickness. After euthanasia, implants for evaluation of degradation were explanted, blotted dry, and weighed (initial wet weight). Next, implants were washed and dried to constant weight at 40°C. Upon reaching constant weight the final dry mass and thickness were determined. The water uptake of the implants was defined as the difference between initial wet weight and final dry weight, relative to its final dry weight.

*Tissue response*: The implants selected for evaluation of tissue response were removed completely from the surrounding skin and subsequently fixed in 4% formaldehyde in PBS (pH 7.4) for 5 days. Next, the samples were washed, dehydrated in graded alcohol solutions, and embedded in glycolmethacrylate (Technovit 7100, Kulzer Histo Technik, Germany). Histological sections were prepared and stained with hematoxylin and eosin, and the tissue inflammatory response was evaluated semi‐quantitatively using light microscopy according to the grading scale explained in **Table** **1**.^[^ ^39^, ^40^, ^41^ ^]^ The images were generated using a Pannoramic 250 FLASH II digital slide scanner (3DHISTECH, Budapest, Hungary).

**Table 1:** Histological grading scale of the tissue response to soft‐tissue implants.

Evaluation	Response	Score
Capsule quantitatively	1‐4 fibroblasts	4
	5‐9 fibroblasts	3
	10‐30 fibroblasts	2
	>30 fibroblasts	1
	Not applicable	0
Capsule qualitatively	Capsule is fibrous, mature, not dense, resembling connective or fat tissue in the non‐injured regions	4
	Capsule tissue is fibrous but immature, showing fibroblasts and little collagen	3
	Capsule tissue is granulous and dense, containing both fibroblasts and many inflammatory cells	2
	Capsule consists of masses of inflammatory cells with little or no signs of connective tissue organization	1
	Cannot be evaluated because of infection or other factors not necessarily related to the material	0
Interface qualitatively	Fibroblasts contact the implant surface without the presence of macrophages or leucocytes	4
	Scattered foci of macrophages and leucocytes are present	3
	One layer of macrophages and leucocytes are present	2
	Multiple layers of macrophages and leucocytes present	1
	Cannot be evaluated because of infection or other factors not necessarily related to the material	0

Adapted from: Link et al.^[^ ^41^ ^]^ and Jansen et al.^[^ ^39^ ^]^

### 2.6 Statistics

The differences in the outcomes between different time points separately for each network and the difference between the three networks at each time point were evaluated by one‐way ANOVA using Bonferoni posthoc analysis (IBM SPSS Statistics 28). In addition, for the differences at time points where no measurements of P(TMC‐*co*‐ε‐CL) 52:48 could be obtained, the differences between the two remaining networks were analyzed by a two‐tailed independent t‐test (IBM SPSS Statistics 28). Results were considered statistically significant when *p* < 0.05.

### 2.7 Results and Discussion

#### 2.7.1 Synthesis and Characterization of PTMC and P(TMC‐co‐ε‐CL) Macromers

Three‐armed PTMC and P(TMC‐*co*‐ε‐CL) macromers, respectively PTMC‐tMA and P(TMC‐*co*‐ε‐CL)‐tMA, were prepared by (co)polymerization of TMC with ε‐CL, and subsequent functionalization with methacrylic anhydride to obtain macromers with methacrylate end groups. The initiator concentration was adjusted to obtain oligomers with an M_n_ of 30 kg mol^−1^. The targeted TMC:ε‐CL molar ratios were 75:25 and 50:50. *M*_n_ of the (co)polymer oligomers, the molar ratios, and the degree of functionalization of the macromers were determined from the ^1^H‐NMR spectra.

The obtained TMC:ε‐CL molar ratios were 74:26 and 52:48. The *M*_n_ of all oligomers was close to the targeted value of 30 kg mol^−1^, and degrees of functionalization were at least 82%. An overview of the results of these analyses is shown in **Table** **2**. In addition, an overview of the ^1^H‐NMR spectra can be found in the supporting information Part B.

**Table 2:** Macromer and network properties as determined by ^1^H‐NMR and DSC.

	Macromers			Networks
	Molar monomer ratio	Mn [kg/mol]	DF [%]	Tg [˚C]
PTMC‐tMA	−	27.5	97	−18
P(TMC‐*co*‐ε‐CL)‐tMA	74:26	32.5	96	−34
	52:48	28.3	82	−46

#### 2.7.2 Characterization of Photo‐crosslinked Networks

The extracted and dried networks were analyzed with regard to their thermal properties. The results of these experiments are shown in Table 2.

For biomedical applications, it is important that the T_g_ is below body temperature. Networks prepared from PTMC homo‐macromers had a T_g_ of approximately −18 ⁰C, comparable to values from the literature.^[^ ^14^, ^27^ ^]^ The networks prepared from the P(TMC‐*co*‐ε‐CL) macromers had low glass transition temperatures, these decreased from −34 to −46 ⁰C with increasing ε‐CL content. This can be expected as the T_g_ of PCL homo‐networks is approximately −60 ⁰C.^[^ ^42^ ^]^ No melting temperatures were observed.

#### 2.7.3 In vitro Degradation of Photo‐crosslinked Networks

In our previous work, in vitro degradation of PTMC homopolymer networks was investigated. While the networks did not degrade in PBS, they did degrade enzymatically by surface erosion using cholesterol esterase, albeit slowly.^[^ ^27^, ^31^, ^43^ ^]^ The mass loss varied between 1.7% and 3.2% with no significant difference between the different networks. However, in vivo it was shown that networks prepared from higher molecular weight macromers (18 and 27 kg mol^−1^) degraded a little faster than those prepared from lower molecular weight macromers (13 kg mol^−1^): from 12 weeks onward this difference was statistically significant.^[^ ^27^ ^]^ After 36 weeks, the networks based on 13, 18, and 27 kg mol^−1^ macromers had a mass loss of 0.5, 3.6, and 2.5%, respectively. In addition, the networks prepared from 18 and 27 kg mol^−1^ macromers showed significant loss of thickness during degradation in vivo, with a decrease of 71 and 92 nm day^−1^, respectively. Therefore, we used the higher molecular weight macromers in the current study.

We hypothesized that networks prepared from copolymers of TMC and ε‐CL would degrade significantly faster than a PTMC homopolymer network. Networks prepared from low molecular weight P(TMC‐*co*‐ε‐CL) degraded by surface erosion^[^ ^31^ ^]^ that is crucial to maintain the material's mechanical strength.^[^ ^44^ ^]^ Preliminary work showed that P(TMC‐*co*‐DLLA) networks degraded by bulk erosion (see the supporting information part A). As we are aiming for surface erosion, in this work we focused on the degradation of P(TMC‐*co*‐ε‐CL)‐based networks, prepared from relatively high molecular weight macromers. A concentration of 20 µg ml^−1^ cholesterol esterase in PBS was chosen as this is the same concentration used in our previous study on PTMC photo‐crosslinked networks,^[^ ^27^ ^]^ as well as in earlier studies on high molecular weight gamma irradiation‐crosslinked PTMC networks.^[^ ^37^ ^]^

**Figure 1:**
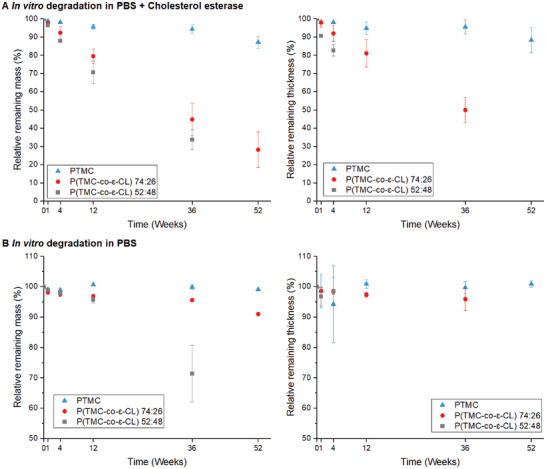
In vitro degradation of PTMC and P(TMC‐*co*‐ε‐CL) networks in PBS containing cholesterol esterase and in PBS. A) shows the relative remaining mass (left) and thickness (right) of the networks in solutions of PBS containing cholesterol esterase. B) shows the relative remaining mass (left) and thickness (right) of the same networks in PBS. Note that the relative remaining thickness of the networks containing 26 and 48 mol% ε‐CL could not be determined after 36 and 4 weeks, respectively. Furthermore, the relative remaining mass of the networks containing 48 mol% ε‐CL could not be determined after week 36. Data is shown as average ± standard deviation (*n* = 3).

A network of the PTMC homopolymer prepared from a similar molecular weight was included as a reference for the degradation. As can be seen from **Figure** **1**A, this network degraded slowly with a remaining mass of 87.2% ± 3.3% after 52 weeks of degradation in PBS containing cholesterol esterase. At 36 weeks, this mass loss was statistically significant (*p* < 0.05). From Figure 1B it can be seen that in PBS, no significant degradation took place.

Both networks prepared from P(TMC‐*co*‐ε‐CL) indeed degraded faster than the PTMC homopolymer network in PBS + cholesterol esterase. At 4 weeks (and onward), this difference was statistically significant. The P(TMC‐*co*‐ε‐CL) 74:26 networks steadily degraded over the 52‐week period with a final remaining mass of 28.2% ± 9.9% in PBS containing cholesterol esterase.

In PBS, the P(TMC‐*co*‐ε‐CL) 74:26 networks showed only limited, albeit statistically significant, degradation with 91.0% ± 0.5% remaining mass after 52 weeks.

**Figure 2:**
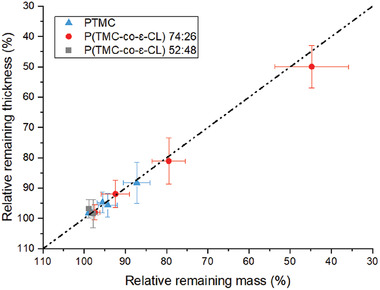
The relationship between the relative remaining mass and the relative remaining thickness of PTMC homo‐ and P(TMC‐*co*‐ε‐CL) copolymer networks during in vitro degradation in PBS containing cholesterol esterase. For all samples, for any given timepoint that both remaining mass and thickness could be measured, the relative remaining mass is plotted versus the relative remaining thickness showing that the relative mass loss correlates to the relative loss in thickness, indicating surface erosion. Data is shown as average ± standard deviation (*n* = 3).

**Figure** **2** shows the relationship between the remaining mass and thickness of the P(TMC‐*co*‐ε‐CL) networks during degradation in PBS containing cholesterol esterase. The direct correspondence of the mass loss with the decrease in thickness over at least 36 weeks indicates that these networks unmistakably degrade by a surface erosion process (at a rate of 0.99 µm day^−1^). This is corroborated by the qualitative assessment of the mechanical properties of these networks as shown in **Table** **3**. At least until 36 weeks, the P(TMC‐*co*‐ε‐CL) 74:26 samples largely maintain their mechanical properties. Only at 52 weeks samples become soft and pliable, although we did not observe any brittleness. Due to the softness, and the tendency of the soft, wet samples to fold upon themselves and stick together, it was impossible to measure the thickness of the samples at 52 weeks. These observations are in line with Chapanian et al. who reported that P(TMC‐*co*‐ε‐CL) networks prepared from macromers of a much lower molecular weight of 8.2 kg mol^−1^ degraded in cholesterol esterase‐containing media by surface erosion.^[^ ^31^ ^]^

**Table 3:** Qualitative assessment of the mechanical stability of PTMC and P(TMC‐*co*‐ε‐CL) networks during incubation in PBS containing cholesterol esterase.

Network	Degradation time [weeks]					
	0	1	4	12	36	52
PTMC	+	+	+	+	+	+
P(TMC‐*co*‐ε‐CL) 74:26	+	+	+	+	+	±
P(TMC‐*co*‐ε‐CL) 52:48	+	+	±	±	−	−

(+) indicates that the mechanical properties were comparable to those at the starting conditions;

(±) indicates that the networks had taken up liquid and had become malleable;

(−) indicates that the networks had become brittle.

The P(TMC‐*co*‐ε‐CL) 52:48 networks degraded faster than the 74:26 networks with a remaining mass of 33.7% ± 5.4% after 36 weeks in PBS containing cholesterol esterase. The former networks had a much larger water uptake, resulting in malleable samples at 4 weeks (see Table 3), with thickness measurements not possible anymore at 12 weeks. Indeed, at 36 weeks samples fell apart upon handling and at 52 weeks the networks were observed to be a “viscous gum”. The condition of the P(TMC‐*co*‐ε‐CL) 52:48 networks in plain PBS at 52 weeks was nearly identical, suggesting that at least for some part bulk erosion takes place in these networks.

These results confirm our hypothesis that TMC and ε‐CL copolymer networks have increased degradation rates compared to networks of PTMC homopolymer. The incorporation of ε‐CL adds ester bonds to the polymer network that can also degrade by non‐enzymatic hydrolysis, whereas PTMC networks consist of carbonate bonds that can only degrade enzymatically. The copolymer networks can thus degrade both enzymatically and hydrolytically, increasing the degradation rate. In addition, increasing the amount of ε‐CL appears to allow for higher water uptake that may further increase the degradation rate.

#### 2.7.4 In vivo Degradation of Photo‐crosslinked Networks

To evaluate the in vivo behavior, PTMC and P(TMC‐*co*‐ε‐CL) networks were implanted subcutaneously in rats. No adverse events with regard to animal welfare were observed postoperatively.

**Figure 3:**
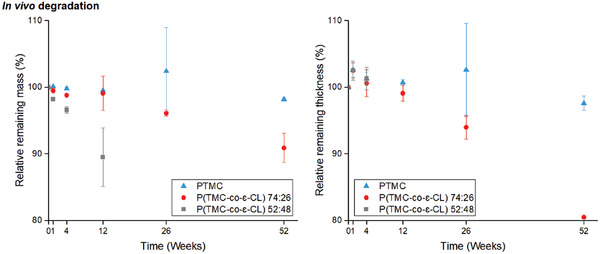
In vivo degradation of PTMC and P(TMC‐*co*‐ε‐CL) networks in rats. The relative remaining mass is shown on the left and the relative remaining thickness is on the right. Note that after 12 and 4 weeks, respectively, the relative remaining mass and thickness of the networks containing 48 mol% ε‐CL could not be determined. Data shown as average ± standard deviation (*n* = 4 except for P(TMC‐*co*‐ε‐CL) 74:26 week 52 (*n* = 1) and P(TMC‐*co*‐ε‐CL) 52:48 week 4 (*n* = 3)).

**Figure 4:**
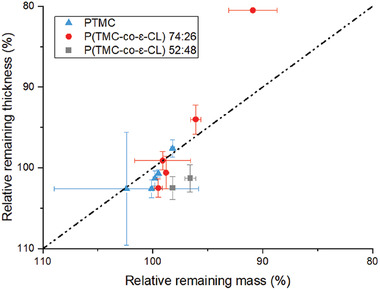
The relationship between the relative remaining mass and the relative remaining thickness of PTMC homo‐ and P(TMC‐*co*‐ε‐CL) copolymer networks during in vivo degradation in rats. For all samples, for any given timepoint that both remaining mass and thickness could be measured, the relative remaining mass is plotted versus the relative remaining thickness showing that the relative mass loss correlates to the relative loss in thickness, indicating surface erosion. Note that for one timepoint of the P(TMC‐*co*‐ε‐CL) 74:26 samples, the thickness of only one sample could be determined. For this sample the standard deviation is not available. Data shown as average ± standard deviation (*n* = 4 except for P(TMC‐*co*‐ε‐CL) 74:26 week 52 (*n* = 1) and P(TMC‐*co*‐ε‐CL) 52:48 week 4 (*n* = 3)).

Compared to previous degradation studies, we prepared polymers with higher molecular weights^[^ ^31^ ^]^ and used longer degradation times. The in vivo degradation of PTMC homopolymer networks was limited and not statistically significant, with a remaining mass of 98.2% ± 0.18% at 52 weeks, see **Figure** **3**. The remaining thickness was very similar with 97.6% ± 1.09% (corresponding to a surface erosion of 33 nm day^−1^), reconfirming the findings of limited surface erosion from our previous study.^[^ ^27^ ^]^ The P(TMC‐*co*‐ε‐CL) 74:26 copolymer networks degraded significantly faster (*p* < 0.05) with a remaining mass of 90.9% ± 2.19% at 52 weeks. The thickness appeared to have decreased more. However, at 52 weeks only 1 sample could be analyzed with regards to the thickness. Nevertheless, up to 26 weeks, the relative loss in thickness was almost similar to the relative loss in mass (see also **Figure** **4**), a strong indication of a surface erosion degradation mechanism. The P(TMC‐*co*‐ε‐CL) 52:48 copolymer networks degraded relatively fast, statistically significant compared to the other two networks after 1 week, with 89.5% ± 4.38% remaining mass at 12 weeks. However, these networks already started to lose their structural integrity, and thickness could not be determined anymore. At 26 and 52 weeks, the network integrity loss was even larger and neither the mass loss nor loss in thickness could properly be determined at these time points.

Even though the in vivo degradation was clearly slower than the in vitro degradation (note the different scales of the y‐axis of Figures 1, 3), the trend in both studies is the same. PTMC homopolymer networks showed only limited in vivo degradation during 52 weeks, evidently via an enzymatic surface erosion mechanism. The P(TMC‐*co*‐ε‐CL) 74:26 copolymer networks degraded at a higher rate via surface erosion, largely maintaining their properties. The P(TMC‐*co*‐ε‐CL) 52:48 copolymer networks degraded even faster, losing their integrity early in the degradation process. This is corroborated by the water uptake of the networks in the in vivo degradation study (see the supporting information part C for full data). While the PTMC homopolymer networks had a limited water uptake of a maximum 2.8% ± 0.5% at 52 weeks that was relatively constant in time, the water uptake increased with increasing Ɛ‐CL content to 11.1% ± 1.5% and 18.6% ± 3.9% for the 74:26 and 52:48 networks respectively. In addition, with each consecutive time point the water uptake of the copolymer networks increased.

##### Tissue Response

**Figure 5:**
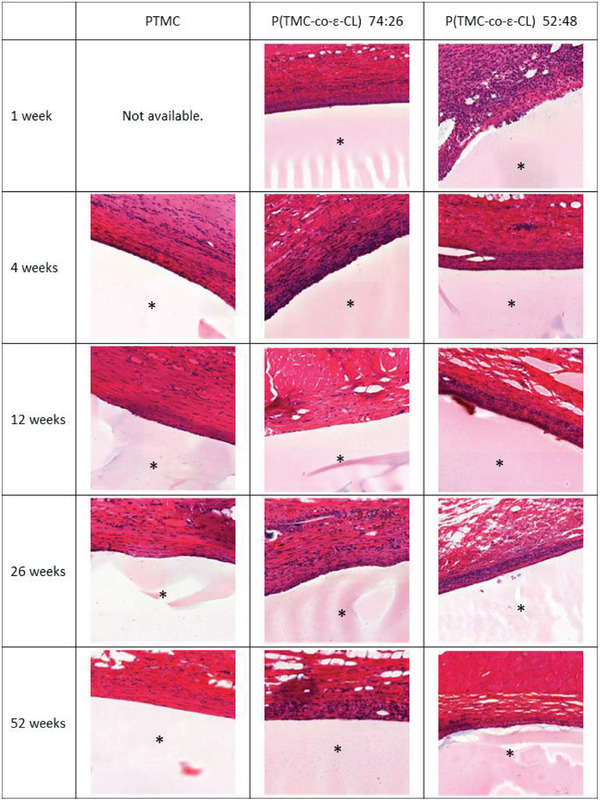
Histological sections of the tissues surrounding the implants were stained at different time points with hematoxylin and eosin. ^*^ = (co)polymer network implant.

The tissue response to the implanted networks was investigated upon hematoxylin and eosin staining and subsequent visual evaluation by semi‐quantitative scoring (see Table 1 for the grading scale, and **Figure** **5** for an overview of the histological sections). In general, the tissue response was characterized by the presence of fibrous tissue, mostly consisting of fibroblasts, although some of the copolymer networks also showed the presence of lymphocytes (the median scores of the grading are shown in **Table** **4**). Encapsulation in a multilayered and organized fibrous tissue was also observed. As such, the tissue response was very similar to our previous study using the homopolymer networks.^[^ ^27^ ^]^ Considering the good tissue tolerance, and surface erosion mechanism shown by the PTMC homopolymer and P(TMC‐*co*‐ε‐CL) 74:26 copolymer networks, it would be justified to use these types of networks for tissue engineering purposes, for instance as scaffolds prepared by stereolithography. The P(TMC‐*co*‐ε‐CL) 52:48 networks lose their mechanical integrity too soon in the degradation process, and therefore these networks are less suited for the aforementioned applications. The deterioration of the mechanical properties of the specimens should be slow enough to allow for tissue ingrowth and organization.^[^ ^26^ ^]^ In particular tissue organization post‐tissue ingrowth has been reported to take much longer than the period of 1–4 weeks characterized by mechanical decline of the P(TMC‐*co*‐Ɛ‐CL) 52:48 networks. This tunable degradation behavior provides researchers with a tool to obtain copolymer networks with the desired degradation rate depending on the intended application.

**Table 4:** Histological grading of the tissue response to subcutaneously implanted PTMC and P(TMC‐*co*‐ε‐CL) copolymer networks at different time points. Data are presented as median scores [range]. A higher score corresponds to a more benign tissue response. See Table 1 for the grading scale.

PTMC				P(TMC‐*co*‐ε‐CL) 74:26			P(TMC‐*co*‐ε‐CL) 52:48		
Time (weeks)	Capsule Quantitatively	Capsule Qualitatively	Interface Qualitatively	Capsule Quantitatively	Capsule Qualitatively	Interface Qualitatively	Capsule Quantitatively	Capsule Qualitatively	Interface Qualitatively
1		No data.		1.0 [1.0;1.0]	3.0 [3.0;3.0]	4.0 [4.0;4.0]	0.0 [0.0;0.0]	1.0 [1.0;1.0]	1.0 [1.0;1.0]
4	1.0 [1.0;1.0]	4.0 [4.0;4.0]	4.0 [4.0;4.0]	1.0 [1.0;1.0]	2.0 [2.0;2.0]	1.0 [1.0;1.0]	1.0 [1.0;2.0]	4.0 [4.0;4.0]	4.0 [4.0;4.0]
12	1.0 [1.0;1.0]	4.0 [4.0;4.0]	4.0 [3.0;4.0]	2.0 [2.0;2.0]	4.0 [4.0;4.0]	4.0 [4.0;4.0]	2.0 [2.0;2.0]	2.0 [2.0;2.0]	1.0 [1.0;2.0]
26	1.5 [1.0;2.0]	4.0 [4.0;4.0]	4.0 [4.0;4.0]	2.0 [1.0;3.0]	4.0 [2.0;4.0]	4.0 [4.0;4.0]	1.5 [1.0;3.0]	2.5 [2.0;4.0]	4.0 [4.0;4.0]
52	2.0 [2.0;2.0]	4.0 [4.0;4.0]	4.0 [4.0;4.0]	2.0 [2.0;2.0]	2.0 [2.0;4.0]	1.0 [1.0;4.0]	2.5 [2.0;3.0]	4.0 [4.0;4.0]	4.0 [4.0;4.0]

## 3 Conclusions

We have prepared biodegradable photo‐crosslinked (co)polymer networks by photo‐crosslinking PTMC and P(TMC‐*co*‐ε‐CL) copolymeric macromers. In vitro degradation experiments showed that PTMC and P(TMC‐*co*‐ε‐CL) copolymer networks degraded at a very low rate in PBS. When cholesterol esterase was added to the medium, in particular the 74:26 copolymer networks were found to degrade by a surface erosion process at an increased rate compared to the PTMC networks. In vivo, while the degradation rates were clearly lower than in vitro, the same trend was observed for the degradation rate of the different networks, with a tissue response characterized by the presence of fibrous tissue, mostly consisting of fibroblasts.

These results show that copolymerization of TMC with ε‐CL in preparing the macromers allowed tuning of the erosion rates of the networks. Therefore, P(TMC‐*co*‐ε‐CL) copolymer networks are interesting materials for tissue engineering or drug delivery applications that require relatively slow degradation by surface erosion.

## Conflict of Interest

The authors declare no conflict of interest.

## Supporting information

Supporting Information

## Data Availability

The data that support the findings of this study are available from the corresponding author upon reasonable request.
