# Supplementary material for: In Vitro and In Vivo Degradation of Photo‐Crosslinked Poly(Trimethylene Carbonate‐co‐ε‐Caprolactone) Networks
Source: Macromol Biosci. 2023 Nov 15;24(3):2300364. doi: 10.1002/mabi.202300364 (PMC13420913; doi:10.1002/mabi.202300364)
Supplement: Supplementary file 1 — Supporting Information [file MABI-24-2300364-s001.pdf]

Macromolecular Bioscience

Supporting Information

for *Macromol. Biosci.*, DOI 10.1002/mabi.202300364

In Vitro and In Vivo Degradation of Photo-Crosslinked Poly(Trimethylene Carbonate-*co*-ϵ-Caprolactone) Networks

Bas van Bochove, Jan J. Rongen, Gerjon Hannink, Jukka V. Seppälä, André A. Poot and Dirk W. Grijpma*

Supporting information

In vitro and *in vivo* degradation of photo-crosslinked poly(trimethylene carbonate-*co*-ε-caprolactone) networks

Bas van Bochove, Jan J. Rongen, Gerjon Hannink, Jukka Seppälä, André A. Poot, Dirk W. Grijpma*

A) Preliminary work on P(TMC-co-DLLA) networks

Experimental:

Synthesis and Characterization of P(TMC-co-DLLA) Macromers

Random three-armed P(TMC-co-DLLA) oligomers were synthesized by ring-opening polymerization of TMC with DLLA using TMP as initiator and $Sn(Oct)_2$ as catalyst. The monomers and initiator were charged in a silanized three-necked flask and polymerized for 3 days at 130 ºC under argon atmosphere. By controlling the monomer to initiator ratio, oligomers with a targeted molar mass (M_n) of 30 kg/mol were prepared. Furthermore, molar ratios of 75:25 and 50:50 TMC:DLLA were targeted. The M_n of the obtained oligomers and molar ratios were determined by ^{1}H-NMR (Varian Inova 400 MHz, Brüker, Germany) using deuterated chloroform solutions.

The oligomers were dissolved in dried DCM (2 ml/g monomer) and subsequently functionalized under argon atmosphere by reaction with methacrylic anhydride (7.5 mol/mol oligomer) in the presence of triethylamine (7.5 mol/mol oligomer) and 0.1 wt% hydroquinone. After 5 days at room temperature, the methacrylate-functionalized oligomers (macromers) were precipitated in cold ethanol and dried under vacuum for 1 week. The degree of functionalization of the macromers was also determined by ^{1}H-NMR.

Preparation and Characterization of Photo-crosslinked Networks

The photo-crosslinked networks were prepared in a UV-crosslinking cabinet (365 nm, 8-10 mW/cm^2, Ultralum, USA). The macromers were mixed with 2 wt% photo-initiator and

subsequently the mixtures were shaped into films by compression molding in a stainless steel mold (100x100x0.5 mm^3) at 70 ^{0}C. The UV cabinet was pre-heated to 70 ^{0}C and the sheets were irradiated for 30 minutes under a nitrogen atmosphere.

Results:

Macromer characterization

The molar ratios and the M_n of the P(TMC-co-DLLA) oligomers were determined by comparing the integral values of the D,L-lactide –CH- peak at δ 5.17 ppm and the TMC $-CH_2-$ peaks at δ 2.05 and 4.24 ppm to the value of the $–CH_3$ peak of the TMP initiator at δ 0.92 ppm. The M_n of the obtained oligomers closely matched the targeted 30 kg/mol and were 30.5 and 31.6 kg/mol. The molar ratios were close to those in the feed with 73:27 and 55:45 TMC:DLLA. The small deviation in the 55:45 macromer may be the result of lactide sublimation during the reaction [1]. The degree of functionalization was >90% for these macromers.

In vitro degradation

The in vitro degradation was performed as described in the paper, with time points 0, 1, 2, 4, 8, 12, 26 weeks.

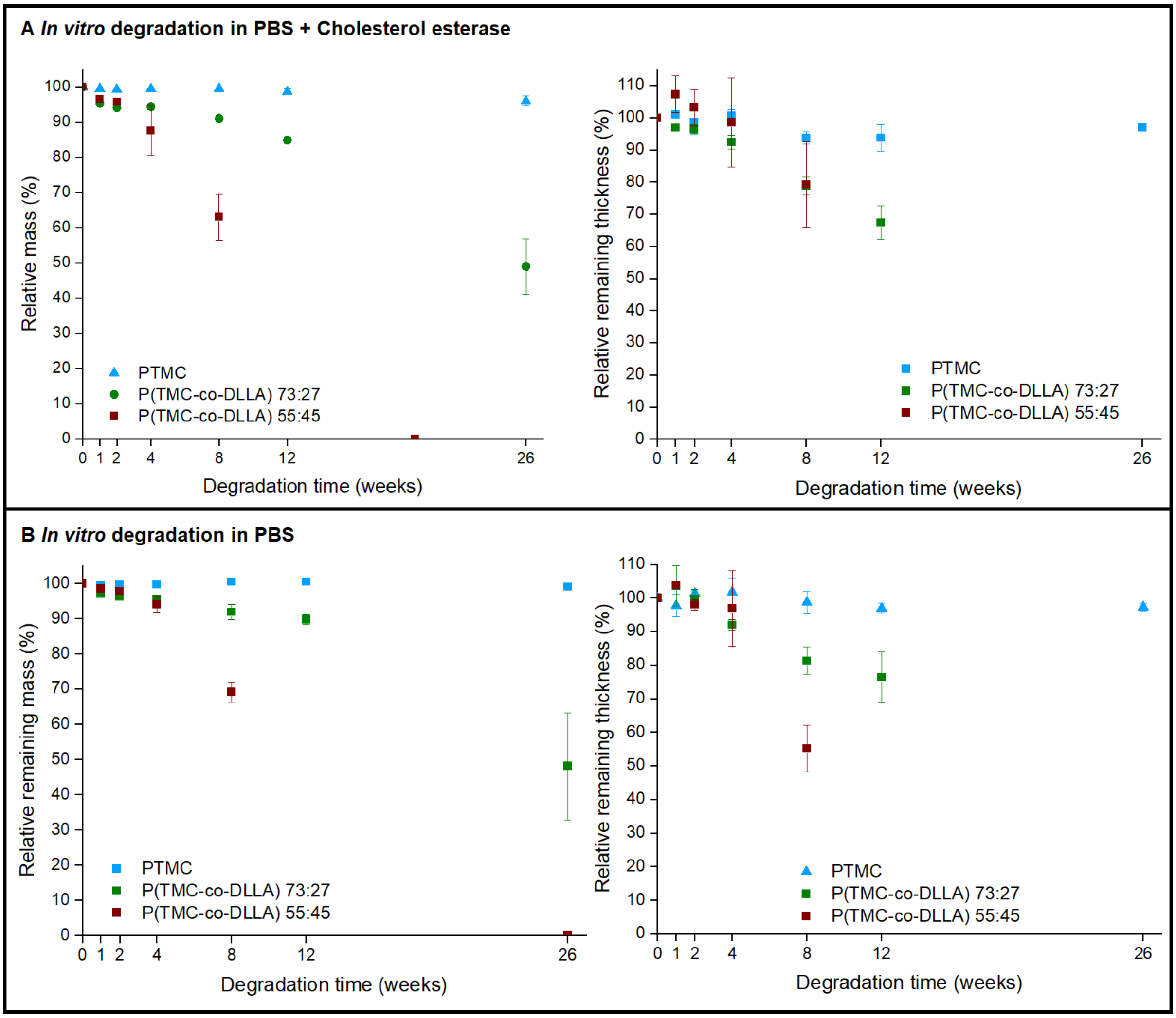

Figure S1. In vitro degradation of PTMC homo- and P(TMC-co-DLLA) copolymer networks in PBS and in PBS containing cholesterol esterase. A) shows the relative remaining mass (left) and thickness (right) of the networks in solutions of PBS containing cholesterol esterase. B) shows the relative remaining mass (left) and thickness (right) of the same networks in PBS. Note that the thickness of the networks containing 27 and 45 mol% DLLA could not be determined after 12 and 8 weeks, respectively. (The data of the PTMC networks is obtained from [2]).

The networks prepared from P(TMC-*co*-DLLA) had higher rates of the loss of mass and thickness than the PTMC homopolymer network as can be seen in Figure S1. Figures S1B shows the relative remaining mass and thickness of the P(TMC-*co*-DLLA) copolymer networks in PBS. In this solution, the homopolymer networks showed almost no loss of mass and thickness. The copolymer network prepared from macromers containing 27 mol% DLLA had a remaining mass of 48.1 ± 15.2% at 26 weeks. Copolymer networks containing 45 mol%

DLLA showed complete mass loss in 26 weeks. The thickness of both DLLA-containing copolymer networks decreased as well. Networks containing 27 mol% DLLA had a remaining thickness of 76.4 ± 7.5% at 12 weeks and networks containing 46 mol% DLLA had a remaining thickness of 55.2 ± 7.0% at 8 weeks. Due to loss in mechanical integrity of the networks, the thickness of the networks after longer periods of degradation could not be determined.

In the cholesterol esterase-containing PBS solution, the homopolymer network had a remaining mass of 96 ± 1.5% at 26 weeks of degradation. The copolymer network containing 27 mol% DLLA had a remaining mass of 49.0 ± 7.9%, approximately equal to the remaining mass in PBS without cholesterol esterase. The copolymer networks prepared from the macromer with 45 mol% DLLA showed complete mass loss at 19 weeks. Both networks also showed a loss of thickness. Networks containing 27 mol% DLLA had a remaining thickness of 67.3 ± 5.3% at 12 weeks and networks containing 45 mol% DLLA had a remaining thickness of 79.2 ±13.2% at 8 weeks. Again, due to loss of mechanical integrity of the networks the thickness of networks could not be determined after longer periods of degradation.

For TMC:DLLA 73:27 networks, incubation in PBS containing cholesterol esterase resulted in a higher loss of mass in the short term (12 weeks) as compared to PBS. After 26 weeks, however, there did not appear to be an effect of the cholesterol esterase. For TMC:DLLA 55:45 networks, incubation in PBS containing cholesterol esterase resulted in an increased mass loss of approximately 7% at 4 weeks compared to PBS. The networks showed complete mass loss in 19 weeks in cholesterol esterase solution, while in PBS the mass loss was complete in 26 weeks.

Figure S2 shows the relationship between the change in mass and thickness during degradation of the PTMC homo- and P(TMC-*co*-DLLA) copolymer network specimens in PBS (Figure S2A) and in PBS containing cholesterol esterase (Figure S2B). For degradation to occur via surface erosion, the relative changes in mass and thickness in time should be approximately equal [3]. The P(TMC-*co*-DLLA) copolymer networks investigated in this study showed simultaneous loss of mass and loss of thickness. Figure S2 indicates that only for time periods of up to approximately 12 weeks, the degradation process shows some characteristics of surface erosion.

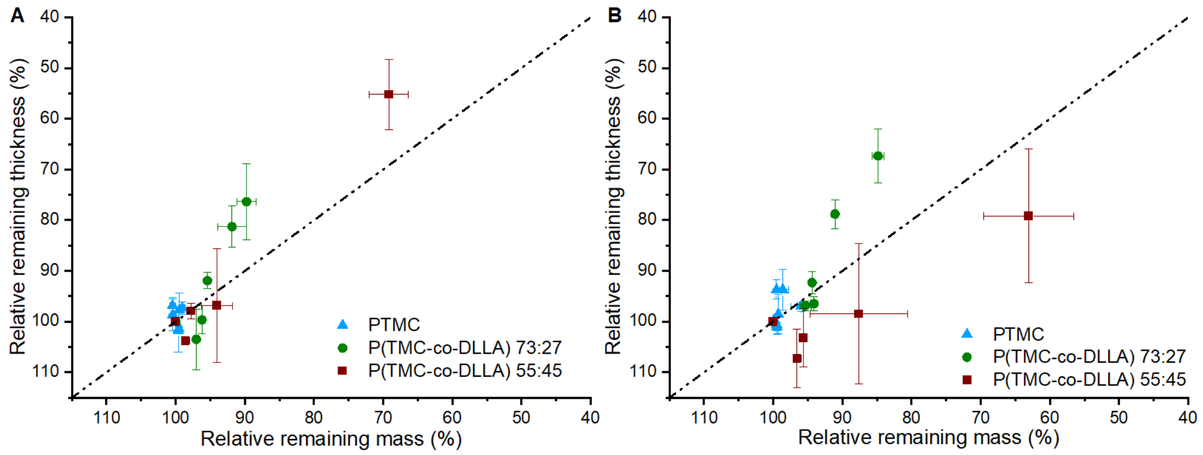

Figure S2. The relationship between the relative remaining mass and the relative remaining thickness of PTMC homo- and P(TMC-co-DLLA) copolymer networks during degradation in: A) PBS and, B) in PBS containing cholesterol esterase. (The data of the PTMC networks are obtained from [2]*).*

It was not possible to determine the thickness of the copolymer networks at later time points than 12 weeks. This can also be seen in the qualitative assessment of the mechanical stability of the networks presented in Table S1.

During the degradation process, the networks first take up liquid becoming gum-like and malleable. In time, the mechanical stability decreases and the specimens become brittle. Depending on the composition, the P(TMC-*co*-DLLA) copolymer networks fragmented at 8 to 26 weeks. Loss of mechanical properties was more noticeable for networks with the higher DLLA content. This loss of mechanical stability was not observed for the PTMC homopolymer networks.

Table S1. Qualitative assessment of the mechanical stability of PTMC and P(TMC-co-DLLA) networks during incubation in PBS containing cholesterol esterase.

(+) indicates that the mechanical properties were comparable to those at the starting conditions, (±) indicates that the networks had taken up liquid and had become malleable, (-) indicates that the networks had become brittle and fragmented upon handling and (--) indicates that the networks had fragmented without handling.

	**Degradation time (weeks)**						
**Network**	**0**	**1**	**2**	**4**	**8**	**12**	**26**
PTMC	+	+	+	+	+	+	+
P(TMC-*co*-DLLA) 73:27	+	+	+	+	±	±	-
P(TMC-*co*-DLLA) 55:45	+	+	+	±	-	--	Complete mass loss

These results suggest that in the first 4 to 8 weeks of degradation in PBS containing cholesterol esterase the P(TMC-*co*-DLLA) copolymer networks degrade via surface erosion. However, at later time points, bulk erosion seems to prevail as the networks fragment catastrophically with significant amounts of material remaining.

The deterioration of the mechanical stability of the P(TMC- *co*-DLLA) copolymer networks in the cholesterol esterase-containing medium, is in agreement with the reported loss of the elasticity modulus, tensile strength and elongation at break of P(TMC-*co*-DLLA) copolymer networks prepared from macromers with lower molecular weights of 8.8 kg/mol *in vivo* which degraded by bulk erosion [4].

B) NMR spectra PTMC and P(TMC-co-ε-CL) oligomers

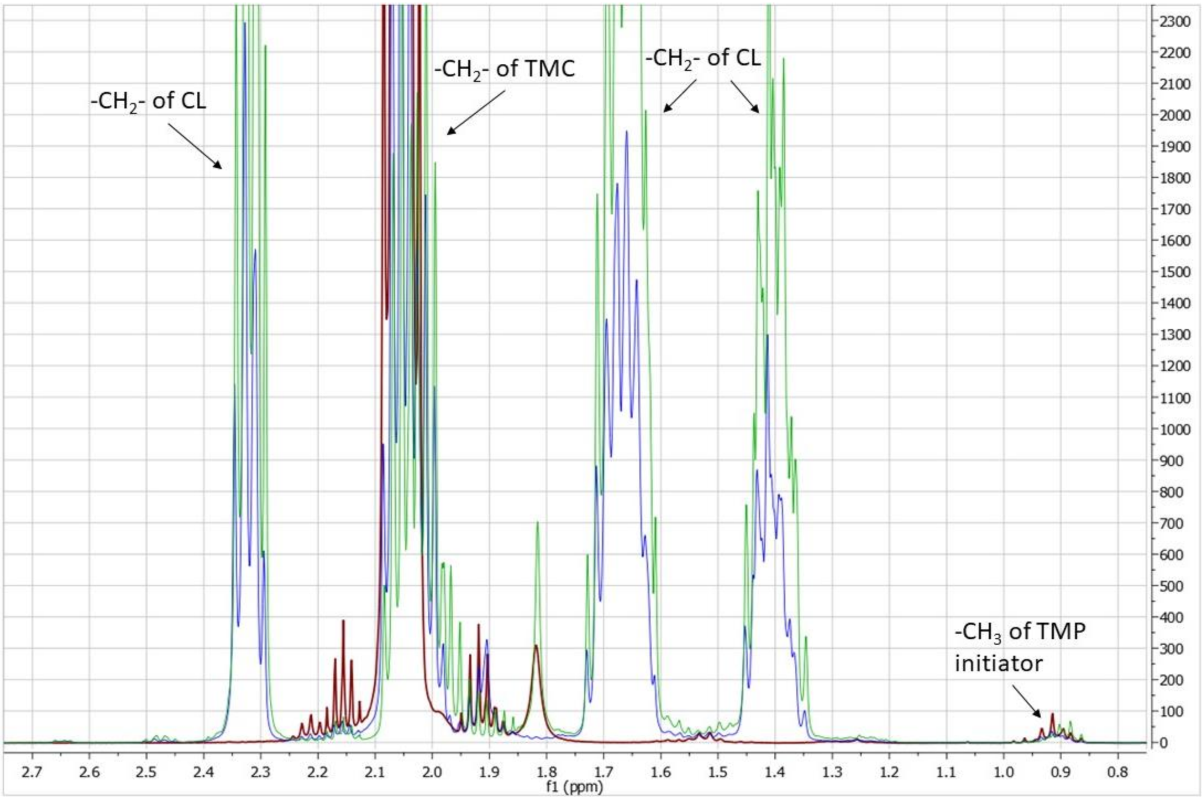

Figure S3. The NMR spectra of the PTMC and P(TMC-co-ε-CL) oligomers used to determine the obtained Mn and molar ratio. In red: PTMC, in blue P(TMC-co-ε-CL) 74:26 and in green P(TMC-co-ε-CL) 52:48. The molar ratio and the Mn of the P(TMC-co-ε-CL) oligomers were determined by comparing the integral values of the ε-CL -CH2- peaks at δ 1.38, 1.65 and 2.31 ppm and the TMC -CH2- peak at δ 2.05 ppm to the value of the –CH3 peak of the TMP initiator at δ 0.92 ppm.

C) Water uptake of implanted PTMC and P(TMC-co-Ɛ-CL) networks

Table S2. Water uptake (%) of implanted PTMC and P(TMC-co-Ɛ-CL) networks.

Time (weeks)	PTMC	P(TMC-co-Ɛ-CL) 74:26	P(TMC-co-Ɛ-CL) 52:48
1	1.9 ± 0.4	3.3 ± 0.7	6.9 ± 0.8
4	2.2 ± 0.4	4.2 ± 0.5	7.1 ± 0.6
12	2.8 ± 0.2	6.5 ± 0.8	18.6 ± 3.9
26	2.5 ± 0.3	7.2 ± 1.1	-[a]
52	2.8 ± 0.5	11.9 ± 1.5	61.5 ± 57.7[b]

[a]No measurements could be done due to samples being stuck to drying cassette after drying.
[b]Measurements done by measuring dry, empty cassettes and then with samples included in wet condition and after drying.

References

[1] S. Sharifi, D. W. Grijpma, *Macromol Biosci* **2012**, *12*, 1423.
[2] J. J. Rongen, B. van Bochove, G. Hannink, D. W. Grijpma, P. Buma, *J Biomed Mater Res A* **2016**, *104*, 2823.
[3] E. Bat, J. A. Plantinga, M. C. Harmsen, M. J. van Luyn, Z. Zhang, D. W. Grijpma, J. Feijen, *Biomacromolecules* **2008**, *9*, 3208.

[4] L. Timbart, M. Y. Tse, S. C. Pang, B. G. Amsden, *Materials* **2010**, *3*, 1156.
